# Prevention of coronary heart disease in people with severe mental illnesses: a qualitative study of patient and professionals' preferences for care

**DOI:** 10.1186/1471-244X-6-16

**Published:** 2006-04-21

**Authors:** Christine A Wright, David PJ Osborn, Irwin Nazareth, Michael B King

**Affiliations:** 1 UCL Department of Mental Health Sciences, Royal Free and University College Medical School (Hampstead Campus), Rowland Hill Street, London, NW3 2PF, UK; 2 UCL Department of Primary Care and Population Sciences, Royal Free and University College Medical School (Hampstead Campus), Rowland Hill Street, London, NW3 2PF, UK

## Abstract

**Background:**

People with severe mental illness (SMI) are at increased risk of developing coronary heart disease (CHD) and there is growing emphasis on the need to monitor their physical health. However, there is little consensus on how services for the primary prevention of CHD should be organised for this patient group. We explored the views of people with SMI and health professionals from primary care and community mental health teams (CMHTs) on how best to provide these services.

**Methods:**

In-depth interviews were conducted with a purposive sample of patients with SMI (n = 31) and staff from primary care (n = 10) and community mental health teams (n = 25) in North Central London. Transcripts of the qualitative interviews were analysed using a 'framework' approach to identify the main themes in opinions regarding various service models.

**Results:**

Cardiovascular risk factors in people with SMI were of concern to participants. However, there was some disagreement about the best way to deliver appropriate care. Although staff felt that primary care should take responsibility for risk factor screening and management, patients favoured CHD screening in their CMHT. Problems with both approaches were identified. These included a lack of familiarity in general practice with SMI and antipsychotic side effects and poor communication of physical health issues to the CMHT. Lack of knowledge regarding CHD risk factor screening and difficulties in interpreting screening results and implementing appropriate interventions exist in secondary care.

**Conclusion:**

Management of physical health care for people with SMI requires complex solutions that cross the primary-secondary care interface. The views expressed by our participants suggest that neither primary nor secondary care services on their own can provide a comprehensive service for all patients. The increased risk of CHD associated with SMI and antipsychotic medications requires flexible solutions with clear lines of responsibility for assessing, communicating and managing CHD risks.

## Background

Cardiovascular disease (CVD) is the leading cause of premature death in England and affects some population groups more than others. In the UK, CVD mortality rates have fallen less than in other developed countries [1], even though the disease is largely preventable and the risk factors are well understood [1]. The UK Government aims to reduce coronary heart disease (CHD) and related disorders by 40% in people under 75 by the year 2010. Their National Service Framework (NSF) for Coronary Heart Disease sets standards for primary prevention, emphasising the need to identify risk and then provide appropriate advice and treatment to reduce this risk [1]. However, it has been argued that there is a need for more research to establish "the clinical, social and ethical acceptability" of the mandated primary prevention interventions [2].

There is growing evidence that people with severe mental illness (SMI) have a two-fold risk of dying from CHD in comparison to the general population [3]. This excess of CHD mortality is due to higher rates of smoking [4], diabetes [5] and dyslipidaemia [5]. It may be further compounded by side effects of antipsychotic medication, which include metabolic side effects such as diabetes mellitus and weight gain [6,7]. The NICE Guidelines for Schizophrenia [8] highlight the need for monitoring the physical health of patients with severe mental illness. Section 1.4.1.3 of the document recommends that this should include the monitoring of cardiovascular risk factors (including checks of blood pressure and lipids) and diabetes, as well as lifestyle factors, such as smoking. The guidelines recognise that some service users may not have regular contact with their GP and recommend that, in such cases, staff in secondary care services should monitor the physical health of their patients.

People with SMI are as likely as the general population to participate in screening for CHD risk factors when this is offered [9]. Although there is a growing emphasis on monitoring the physical health of people with SMI [6-8], especially when prescribing certain atypical antipsychotics, there is little evidence to guide the development of services for screening for CHD risk factors such as diabetes and dyslipidaemia. There is little consensus on how primary prevention of CHD risk should be organised, who is best placed to undertake this task and what is required to support screening. There are no data on the views of general practice and community mental health team (CMHT) staff or service users regarding the acceptability of screening for CHD risk factors. Obstacles to delivery of CHD prevention in SMI remain unexplored and we know little about patients' preferences. The role of primary and secondary care in primary prevention of CHD is poorly defined. Although CHD prevention is traditionally the remit of primary care, some patients are in more frequent contact with CMHTs. Antipsychotic agents associated with weight gain and possible metabolic disturbance are usually initiated and monitored in secondary care.

We aimed to elicit the views of service users (people with SMI) and professionals (i.e. primary care and community mental health staff) on screening for CHD risk factors and interventions for primary prevention of CHD. We sought opinions regarding existing practice, obstacles to accessing care, preferred setting and different service models.

The qualitative study was a component of a wider research programme which aimed to develop appropriate interventions to enhance primary prevention of CHD in people with SMI. The other components of the programme have included a systematic review of the literature on CHD mortality, morbidity and risk factors in SMI; a secondary analysis of CHD mortality and morbidity data on a large national cohort of people with and without SMI, using the UK General Practice Research Database (GPRD); and a large questionnaire survey of service users and health professionals to explore a wider range of health service issues related to the management of CHD in SMI.

## Methods

We recruited participants from Central North London, including staff and service users from CMHTs and general practitioners (GPs) and practice nurses. We contacted a sub-sample of local CMHT and GP practice managers to inform them about the study. Having received the manager's agreement, detailed information sheets were circulated to individual team members with an invitation to take part in an interview. We purposively recruited CMHT staff from a range of disciplines to include community psychiatric nurses, social workers, occupational therapists, consultant and trainee psychiatrists, as well as general practitioners and practice nurses.

Service users were invited via their consultant psychiatrists or CMHT workers. Adults (age 18 to 65) with a diagnosis of SMI (i.e. schizophrenia, schizoaffective disorder, bipolar affective disorder or other non-organic psychotic illness) were eligible to participate in the interviews. Service users were excluded if their current mental and/or physical health was such that it would prevent them from giving informed consent or participating in an interview. Where a participant's first language was not English, we were able to offer the services of an interpreter at the interview. Service users were not excluded if they already had a diagnosis of CHD.

Since service users were approached via their CMHT care coordinators or consultant psychiatrists, none were acutely unwell. They were therefore able to understand information about the research, weigh up the risks and benefits of being involved and make an informed choice about whether to take part in an interview. As well as receiving a detailed information sheet, service users and staff had the opportunity to ask questions about the study before agreeing to take part. Written and verbal informed consent was obtained from all participants. The research was carried out in accordance with the local research ethics committee standards and the Helsinki declaration.

We sought health professionals' and service users' opinions about primary prevention of heart disease in people with SMI through individual face-to-face interviews. Each interview was guided by a semi-structured schedule, with initial open-ended questions to ascertain the participant's general view or preference on each issue followed by more in-depth or 'probing' questions to explore the reasons underlying the view they had expressed. Where necessary, prompts were used to encourage the interviewee to reflect on key issues, if they had not raised these spontaneously. At the end of the interview, participants were given the opportunity to add any further views or comments that they felt were important but which had not been covered in the interview schedule. A semi-structured approach was employed to ensure that we accessed and explored participants' preferences in relation to the key issues relevant to service development whilst still allowing the participant some freedom to express their views on those key issues as fully as they wished. There was scope within the schedule for the ordering of the questions to be flexible where this aided the flow of the discussion.

The questions were adapted to be relevant to primary care staff, CMHT staff or people with SMI. The interviews explored views on physical health care, the most appropriate setting for screening and management, preferences for different models in secondary and primary care, perceived obstacles and problems with different models of care, training needs and the willingness to deliver or receive such care. We defined CHD screening as including routine assessment of smoking status, elicitation of existing history of diabetes, total and HDL cholesterol, random glucose, body mass index and ECG (where relevant), as well as an assessment of diet and exercise. We explored views on the following interventions: smoking cessation; weight reduction; diet and exercise advice; and drug treatment of hypertension, diabetes and dyslipidaemia.

Having heard participants' views on the primary care and secondary care models, we then introduced the idea of a specialist nurse-led model (if this had not already been suggested spontaneously by the participant). In this model, a physical health nurse attached to the CMHT would liaise with primary care teams and CMHTs to ensure that screening is regularly offered to clients with SMI. A "solo-practitioner" model has been suggested as a means of bridging the gap between primary and mental health services, thus helping to overcome some of the barriers that exist to adequate medical and preventive care for people with SMI [10]. Furthermore, there has been a recent move within the National Health Service (NHS) towards specialist nurse-led services (e.g. in the fields of cancer and diabetes care). Opinions were sought on the general idea of a nurse-led service, without being prescriptive as to the precise role such a specialist nurse might adopt.

Patient interviews lasted 20–30 minutes and those with staff 40–45 minutes. All participants were reimbursed ~20 for their time and expenses. All interviews were audio-taped and transcribed by CAW. The interview transcripts were analysed thematically using a 'framework' approach [11]. The thematic framework was guided by the key topics covered in the interview schedules, for instance attitudes to CHD prevention work, where CHD screening should take place, and what might be the main obstacles. The data were charted using Excel 2002. Transcripts were reviewed independently by two members of the research team (CAW, DPJO) to identify common themes and opinions emerging from participants' responses to each of the key topics. Opinions regarding each theme were extracted by each reviewer until saturation point had been reached where no further different opinions were forthcoming. These themes were agreed by consensus, with all the researchers agreeing the final interpretations.

## Results

We interviewed 31 patients with SMI (18 males and 13 females), aged between 28 and 67 years. The psychiatric diagnoses were schizophrenia (n = 15), bipolar affective disorder (n = 12) or schizoaffective disorder (n = 4) and the majority were unable to work due to long-term sickness or disability (n = 20). We also interviewed 35 health professionals (14 males and 21 females), aged between 24 and 57 years. Ten staff worked in primary care and 25 in CMHTs. They included community mental health nurses (n = 10), social workers (n = 7), occupational therapists (n = 1), psychiatrists (3 consultants, 2 registrars and 2 senior house officers) and GPs (n = 8) and practice nurses (n = 2).

### Importance of targeted CHD screening

There was a consensus view that screening for CHD risk factors in people with SMI was an important priority for health service resources, for which a range of reasons were offered (see Table 1). People with SMI and the professionals were aware of the high prevalence of risk factors for CHD. Professionals from all disciplines expressed concern that services neglect the physical health of people with SMI. Service users feared that their physical health needs often "fell through the net". Concerns about the adverse effects of atypical antipsychotic medication were repeatedly articulated by staff and users.

**Table 1:** Views on the importance of CHD risk factor screening and obstacles to its success

	View expressed by *			
**Reasons cited for the importance of CHD screening in SMI**	**CMHT**	**GP**	**SU**	**N**
• There is a high prevalence of smoking and weight problems among their own caseload with SMI				23
• Physical health is often neglected by services due to their focus on clients' mental health problems				20
• Side effects of antipsychotics, e.g. weight gain and metabolic				14
• Physical health can be neglected due to clients' poor motivation and social isolation – they need extra help and encouragement with this				13
• Research evidence indicates people with SMI are a high risk group for CHD				9
• Knowing about one's personal risk for CHD would enable clients to take timely preventative action (e.g. to make lifestyle changes)				8
• Regular screening would allay client's fears about their physical health				7
• Clients are aware of their own risk factors for CHD, especially smoking, family history, diet and weight				6
• Screening should be offered to everyone, regardless of SMI diagnosis				3
• Recent experience of clients with SMI dying due to undetected CHD				2
• The stress of having SMI may adversely affect the heart				2
• People with SMI are harder to engage and so need more assertive screening				2
• It is important for staff to recognise risk and be able to interpret any new physical symptoms as organic rather than psychological in nature				1
				
**Perceived obstacles to/negative views of CHD screening**				
• Lack of appropriate resources in existing services – e.g. time, trained staff				18
• Anticipation of low uptake rates by patients with SMI				17
• Perceived difficulty in making lifestyle changes amongst people with SMI, even if risk CHD factors are identified				15
• Patients dislike having blood tests				12
• Lack of funding for CHD screening services or it not being seen as a priority by Trust management				12
• A screening offer might be viewed as interference in patients' lives – they may feel defensive, anxious or paranoid				7
• Stigma: a perception that services such as smoking cessation can't deal with people with SMI				4
• CMHT services already "squeezed"				4
• Staff resistance to more changes in their role – CHD screening would be moving too far away from their mental health role				4
• Poor communication of results between primary and secondary care				3
• Lack of appropriate services to refer patients to if risk factors are identified – e.g. long waiting lists, narrow referral criteria, group sessions				3
• It would not be cost effective to screen all SMI patients, only those in high risk groups e.g. overweight				2
• Prior experience of low attendance when routine screening appointments were offered to people with SMI in line with the new GP contract				1

* Note: Tick-boxes indicate which group(s) of participants expressed the view: CMHT = staff from community mental health team; GP = staff from general practice; SU = service users. Numbers (N) indicate the prevalence of each view within the total sample.

I think it's very important because I think they [SMI] do have significant problems in this area, partly because of their lifestyle and partly because many of the drugs that they're taking, especially the people on antipsychotics ... atypical antipsychotics, you know, cause weight gain and problems with blood pressure, some of the antidepressants, so, you know ... the medication they're on predisposes them to risk factors for cardiac disease and diabetes and weight gain and dyslipidaemia ... and I don't think we're doing enough to adequately attend to those problems in this population (Consultant Psychiatrist 21).

I think it [check-ups] would be a great idea. I think actually ... not just a good idea, I think it's necessary. Because otherwise they [people with SMI] will just fall through the net ... when the only time they can get a medical check-up is when they have a psychotic episode and they're brought into hospital (Service User 17).

Many respondents expressed concerns that CHD prevention would be very difficult in people with SMI. The obstacles included low uptake rates, high current workload within the CMHT and poor communication between GPs and the CMHT (see Table 1).

Some of our mental health reviews that we're expected to do ... getting people (with SMI) to actually turn up is very difficult (General Practitioner 3).

### Motivation and ability of staff to deliver primary prevention of CHD in SMI

Professionals expressed various attitudes to incorporating CHD screening into their professional role. General practice staff stressed this was already a core component of their work and expected to take on this role in people with SMI. The UK primary care GMS contract requires practices to maintain an SMI register and provide an annual physical health review to clients with SMI. However, some primary care staff identified lack of experience with SMI and unfamiliarity with the CHD-related side effects of some antipsychotic medications as obstacles. One practice nurse stated that it was uncomfortable explaining that individuals had been invited for a physical check-up due to their inclusion on the practice SMI list.

I sort of felt a bit uncomfortable, because they came in and said, "I've got this letter" [about screening appointment] ... and so you sort of skirt around it because you don't want to say, "Oh, this is because you've got mental health problems and you don't come to screening" (Practice Nurse 04).

This unease about maintaining specific lists of people with SMI was repeated by several staff and service users who felt such lists or indeed special clinics were stigmatising or even "ghettoising". However some service users (and staff) voiced an opposing view, stating that special clinics for people with SMI gave them confidence that their mental health problems would be understood.

I think I would appreciate a separate service for mentally ill people, if I was going to my GP. Because I have a mental illness, I sometimes ask too many questions and I feel a bit sort of anxious about seeing the doctor whereas, if the GP knew that I was mentally ill, he'd be more patient with me if I was asking questions (Service User 10).

It would be stigmatising in a way, but I think these people are stigmatised anyway. And I think probably people would get lost in a more general clinic. That's just off the top of my head. I could give you some reasons for both [special vs. general clinics] ... but I think really, if we are going to take people's physical health more seriously, then I think probably to have dedicated services ... a dedicated clinic (Community Psychiatric Nurse 06).

Psychiatric staff rarely included formal assessment of CHD risk factors in their routine practice although some did discuss diet and exercise with patients. They expressed more diverse views on their role in performing this task than the primary care staff. Some viewed physical health care as an essential part of their work and emphasised the importance of an overall holistic approach to their patient's problems.

I think it's holistic. Like I said, it's the whole package, isn't it? You can't just ignore people's physical health and concentrate on their minds. I guess, as a social worker, we're trained to kind of see the whole package, rather than separate little conditions ... or physical health and mental health being split into two (Social Worker 02).

Other CMHT staff felt that physical health was the remit of primary care and that it would be an inappropriate use of their skills and time. They felt responsibility for screening would blur their professional roles. Moreover, they lacked the expertise and resources to provide an adequate primary prevention service for CHD.

I'm all for an holistic approach but ... if I was to speak truthfully ... I'm up to my limit really with mental health ... you know, so it is difficult to even think about some of these other issues because you've got so much else to focus on ... I mean, not just the mental health but the ... all the other stuff that at different levels ... you know, the social aspects of people's lives and their housing problems and their money problems and such-like (Community Psychiatric Nurse 06).

This diversity of this opinion was apparent across the professional disciplines, with some psychiatrists, nurses and social workers expressing a keen desire to include CHD screening in their work. Similarly there were professionals from all disciplines who were against conducting this work.

### Views of primary versus secondary care service models

Most CMHT and primary care staff initially responded that screening would be best performed by primary care services. They described the benefits of a system that is already established to provide screening and relevant interventions for risk factors that are detected.

I don't see why they [SMI] should be treated in a different setting from other patients with the same condition, really. And I think that primary care would be the people who would know better than we would how to manage these things and then would be monitoring them in the longer-term. I think whoever is diagnosing them should really be managing the conditions as well (Consultant Psychiatrist 07).

Preference for a service for the primary prevention of CHD in *secondary care *services was mentioned more frequently by people with SMI, often because they felt more comfortable engaging with mental health staff.

Since it's geared towards mentally ill people, I think that it should be done, if possible, within the mental health service. I say that because, to me, I feel happier coming here to talk about my problem ... my mental health problems ... with a specialist, rather than going to a GP, who's fine but firstly they're not a specialist and secondly they haven't really got much time (Service User 14).

When staff was asked to consider the benefits and disadvantages of both models in turn, a more complex picture emerged. A variety of problems with the primary care model were forthcoming (see Table 2). Poor uptake of screening, lack of engagement of SMI patients with GPs, poor communication of results from GPs to the CMHT and lack of knowledge among GPs regarding the relationship between antipsychotic medication and CHD risk were common concerns.

**Table 2:** Primary care model for CHD risk factor screening in SMI: Advantages and disadvantages

	View expressed by *			
**Perceived advantages of delivering CHD screening in primary care**	**CMHT**	**GP**	**SU**	**N**
• GPs possess medical expertise in CHD screening and can provide appropriate management of results				14
• It is more normalising/less stigmatising to attend primary care for screening				13
• Patients attend the GP practice regularly to pick up their prescriptions, which would offer the opportunity for screening				7
• Clinical systems and equipment are all in place to provide screening				6
• Patients have better links, trust and a longer history with their GP, which would enhance the uptake of screening				6
• GPs can access other relevant physical health services more easily than the CMHT – they are the 'gate keepers'				6
• Geographically, GPs are often closer than the CMHT				6
• It would allow the CMHT to focus on mental health issues				2
• It would engage clients with their GP and encourage people with SMI to be less reliant on mental health services				2
				
**Perceived disadvantages**				
• Lack of resources to provide this service due to GP's high workload				17
• High rates of non-attendance to general practice screening				8
• Specialist SMI screening clinics in primary care could be stigmatising				8
• Communication from primary to secondary care is very rare so psychiatrists won't receive screening results to inform prescribing				7
• Some patients get anxious about attending primary care and the more severely ill are often not in contact – they will not receive screening				7
• Some GPs may be negative towards or disinterested in people with SMI and offer a poor service to them – it could be a 'patchy' service				5
• Some GP staff may lack confidence in working with people with SMI				4
• It maintains a split between physical health (at GP) and mental health (in psychiatry), preventing the person from being seen holistically				1
• Lack of specialist knowledge of possible metabolic effects of antipsychotics amongst GPs				1
• If screening revealed a possible adverse effect of antipsychotics, such as diabetes, GP may lack the confidence to alter the antipsychotic				1
• It can be difficult to get an appointment with the GP				1

* Note: Tick-boxes indicate which group(s) of participants expressed the view: CMHT = staff from community mental health team; GP = staff from general practice; SU = service users. Numbers (N) indicate the prevalence of each view within the total sample.

This complexity was increasingly apparent when participants were asked to consider the pros and cons of a CMHT based model of screening (see Table 3). Many people – who had initially stated that primary care was the best setting for primary prevention of CHD – began to identify disadvantages to the primary care model and advantages to the secondary care model which had not been immediately obvious. The benefits of the CMHT providing such a service included having access to users who rarely visit their GP and leaving the responsibility of early prevention of CHD in the hands of those who managed their antipsychotic medication.

**Table 3:** Secondary care screening model: Advantages and disadvantages

	View expressed by *			
**Perceived advantages of delivering screening in secondary care**	**CMHT**	**GP**	**SU**	**N**
• CMHT staff have a better rapport and understanding of people with SMI				12
• CMHT has better access to and knowledge of people with SMI				8
• The CMHT setting and workers are less threatening for patients than the GP environment and easier to trust – this might reduce the non-attendance rates				6
• CMHT staff can access patients in a greater variety of settings, thus enhancing the uptake of screening				6
• It promotes a more holistic model of care – 'not just a prescription'				4
• It is better to unite clients' physical and mental health care in one place				3
• CMHT staff are more experienced than GPs in working assertively with people with SMI				3
• If the CHD risk factors are linked to having SMI, then the CMHT should take responsibility for screening				3
• Psychiatrists prescribe the antipsychotics which require risk factor screening				2
• CMHT workers have more time and can offer longer appointments				2
• It would allow CMHT staff to develop new skills				1
• There are shorter waiting times at CMHT compared to the GP				1
				
**Perceived disadvantages**				
• The CMHT workload is already high – they lack the time for extra responsibilities				19
• Lack of skills and knowledge required for screening amongst care coordinators, especially those without nursing or medical training				12
• Lack of appropriate facilities – e.g. equipment, clinical rooms, access to blood results in community settings				9
• Unwillingness of CMHT staff to take on extra roles				8
• Lack of medical expertise in the CMHT regarding appropriate interventions if screening results are positive – care will either be inferior or simply result in re-referral to primary care.				5
• It blurs the role of the CMHT				5
• Some service users mistrust psychiatric services and don't want their involvement				4
• CMHTs only see the most severely mentally ill people, so some patients will be overlooked				3
• It would be stigmatising (not normalising) to have separate services for people with SMI				3
• Patients like to keep their mental health and physical health separate				3
• Mental health meetings such as Care Programme Approach meetings are inappropriate settings for screening				2
• It would cause stress for CMHT staff who might feel to blame if CHD morbidity was undetected				2
• Lack of continuity with CMHT staff – they tend to come and go more often than GP staff				2
• CMHT bases are less accessible than GPs geographically				1

* Note: Tick-boxes indicate which group(s) of participants expressed the view: CMHT = staff from community mental health team; GP = staff from general practice; SU = service users. Numbers (N) indicate the prevalence of each view within the total sample.

Psychiatrists don't really often go below the neck ... in terms of the things they look out for and, if they're going to be handing out medicines, such as Risperidone or Olanzapine or Quetiapine, which of course make people fatter ... makes them diabetic ... then I think that's a responsibility they have to take – of being able to screen individuals for coronary heart disease and do something about it (General Practitioner 10).

The main disadvantages were being overworked and possibly unfamiliar with blood results and appropriate interventions such as anti-hypertensives, lipid lowering medication or diagnosis of diabetes and its management.

I think the risk would be that you're just filling in boxes and maybe they'd come to the clinic, someone would check their blood pressure, take the blood test, do the height and weight ... and you'd tick the boxes and say, "Right, we've done it" but then nothing really would be done about it. And I think it would be the next step about who would take the responsibility of saying, "Well, you've got high blood pressure" or "Your cholesterol is too high. We need to do something about it" ... and who then would go on to do that. What would happen? (Psychiatrist 23).

### Shared care services or a service led by additional nursing staff

Having discussed the pros and cons of primary and secondary care screening, participants were asked to consider the best way forward. Several staff spontaneously suggested the need for improved collaboration between primary and secondary care services to ensure that screening was provided coherently and comprehensively.

I suppose we've been thinking a bit 'either-or' but, in a way, it's going to have to be 'both-and' because there's ... you know, two-thirds of mental health we don't see [in mental health services] anyway ... there are going to be some patients who don't make it to us and there are going to be some patients that we see who don't really make it to the GP. So, if you want to cover the entire spectrum, I think you need to do both (Psychiatrist 24).

However, there were still drawbacks to such models including issues about confusion of roles, who was actually taking responsibility for provision of the screening and for acting on any abnormal findings. Poor communication, especially from primary to secondary care, was again felt to be a major flaw in joint (or collaborative) working. Many participants agreed that one person should take overall responsibility to ensure adequate screening for risk factors of CHD – be it primary or secondary care – depending on patient, professional and local service characteristics. When a specialist nurse-led service was suggested to participants who had not raised it spontaneously, they usually endorsed it. The perceived benefits were its flexibility, greater accessibility for patients, clarity of roles and ensuring good liaison between primary and secondary care, irrespective of individual patient or staff preferences for delivery of screening (see Table 4).

**Table 4:** Views regarding a specialist nurse model for providing CHD screening in SMI

	View expressed by *			
**Perceived advantages**	**CMHT**	**GP**	**SU**	**N**
• There would be greater accessibility to screening for patients				5
• It would introduce more flexibility into a system which otherwise only suits certain patient profiles				4
• The nurse would provide specialist cross-disciplinary knowledge in a complex area that few CMHT staff or GPs feel wholly confident in.				4
• This model has worked successfully in other areas e.g. offering HIV testing in a drug dependency unit; a hepatitis nurse attached to a community drug team				3
• A nurse could bridge the existing gaps between primary and secondary care				3
• It would prevent the burden falling on already overworked CMHT workers and allow them to concentrate on their "traditional [mental health] work".				3
• Mobile services are also successful e.g. breast screening, needle exchanges				3
• It would facilitate communication, allow monitoring of the service and improve liaison between different parts of the service.				3
• It would allow a trusting ongoing clinical relationship to be established with the specialist nurse who knows about physical health issues				2
• The nurse could take on additional roles e.g. running interventions, groups, prescribing				1
• Someone needs to take specific responsibility to ensure that screening does happen				1
• One specialist nurse could be employed across whole Primary Care Trusts				1
				
**Perceived disadvantages**				
• There might be too much or (in the view of a different participant) too little work for the specialist nurse to provide				7
• It would be an expensive option and thus is unlikely to be prioritised or commissioned – non-attendance rates may be too high to justify the cost				6
• It adds another person into the health service equation and complicates it				2
• It might encourage further dependence on the service by people with SMI, rather than them accessing their GP like everybody else				1
• It may make patients link physical side effects to their antipsychotic medication, encouraging cessation of treatment				1
• It may create suspicion when SMI patients feel "singled out" for a special service				1

* Note: Tick-boxes indicate which group(s) of participants expressed the view: CMHT = staff from community mental health team; GP = staff from general practice; SU = service users. Numbers (N) indicate the prevalence of each view within the total sample.

I think it's always good to have somebody whose kind of responsibility it is to make sure that things happen... I mean, if there was funding. That person wouldn't necessarily do it all but they would kind of have an overall responsibility for making sure that it, you know, happened and coming round to the practices and saying, "What are you doing?" and checking our records, or whatever, to find out (General Practitioner 01).

I think that [the specialist nurse model] would be best of all, because of its flexibility and because of the recognition that you really need to try and catch people in different settings. In fact, there probably isn't a one-size-fits-all scenario to offer this service really (Consultant Psychiatrist 25).

However, despite their enthusiasm, respondents were not optimistic about the likelihood of health service funding being available for this option.

## Discussion

The views expressed by our participants suggest that the provision of comprehensive screening for CHD risk factors in people with SMI is seen as a health service priority by many patients, CMHT staff – including psychiatrists – and general practice doctors and nurses. The provision of such services, however, is likely to require complex models of care that cross primary and secondary care services and allow for flexibility in staff and patient motivation, and preferences for services, with an emphasis on communication between different elements of the service.

Prioritisation of physical health in SMI is partly explained by observation of CHD risk factors and mortality in people with SMI and growing concern regarding the adverse effects of antipsychotic medications. The spontaneous reaction of CMHT and GP staff in our sample was often that CHD screening should remain the remit of primary care services where it has traditionally been located. Service users recruited from CMHTs were more divided in their views and many said they would prefer to receive primary prevention of CHD as a service in their CMHT. Despite this enthusiasm from patients, professionals felt that the provision of CHD screening and relevant interventions would require more assertive effort in this patient group.

Our participants' opinions indicate that provision of a comprehensive CHD screening service is likely to require a complex approach that crosses the primary-secondary care interface. Many felt that models located solely in primary or secondary care would be unlikely to benefit all patients. Individual patient factors (such as preference for primary or secondary care settings and teams), individual staff factors (knowledge, workload, motivation and interest in mental illness or CHD screening) and communication factors could produce inconsistent levels of CHD-related care in either primary or secondary care settings. Many of our participants therefore concluded that a service led by at least one person liaising between primary and secondary care was the best solution.

The possibility of liaison across the primary secondary interface is not a part of the growing body of recommendations regarding the provision of physical health care and screening for CHD risk factors such as diabetes, smoking and dyslipidaemia in people with SMI. International guidelines on the monitoring of CHD risk in schizophrenia suggest that screening for lipids and diabetes should normally take place in primary care, unless the patient is not in contact with such services [7,8]. In the UK, the new GP contract places responsibility for an annual physical health check upon primary care, and remunerates practitioners if targets are met. However, the required content of such check-ups does not automatically include diabetic status or lipids. Furthermore there is no requirement to communicate such CHD risk results back to secondary care. The service users and professionals we interviewed felt that this primary care model generated many problems. SMI lists tended to be stigmatising, essential clinical information was unlikely to be available to the psychiatrist managing the antipsychotic medications, and many participants did not attend for routine appointments in primary care. Some service users also cited difficulty accessing primary care appointments.

Other international commentaries have suggested that screening should become the remit of secondary care. Since psychiatrists and CMHT staff are in more frequent contact with people with SMI and since they prescribe antipsychotic medication, it is stated that they should be more familiar with CHD risk factors [6,7,12]. This view was supported by many of our participants but it was also evident that placing such responsibility on all CMHT staff is not universally acceptable and would generate many difficulties. Staff would require training in interpretation of CHD risk factors and their management. Whilst some CMHT staff welcomed the opportunity to expand their skills, others felt that such training would detract from their mental health and social work skills, and might even lead to people with SMI receiving an inefficient or inadequate service for the primary prevention of CHD, compared to the services provided to the general population in primary care.

CHD screening requires specialist knowledge on interpretation of blood results and a knowledge of which interventions are required within what timeframe, for different magnitudes of CHD risk factors. This necessitates repeated measurement of risk factors to assess the effect of the interventions. These more complex components of primary prevention of CHD cannot be expected of CMHTs, and therefore raise important questions about the level of CMHT involvement that is appropriate. However, the fact remains that while GPs are monitoring raised CHD risk, psychiatrists who possess knowledge regarding antipsychotics need to be aware of lipid, diabetic or other CHD complications which would influence the risk/benefit equation sufficiently to change their choice of prescription. High rates of CHD risk factors in this group of patients necessitate assertive efforts to offer screening and relevant interventions for people with SMI. Our research demonstrates that the interface between primary care and CMHTs potentially creates multiple pitfalls to providing such services. If primary prevention services are to be successful, they will need to overcome a range of obstacles. Some of the barriers our participants identified are specific to people with SMI, such as experience or perception of stigma regarding their mental health. Others are non-specific, such as dislike of blood tests. Comprehensive services must overcome diverse training needs at primary and secondary care levels, as well as the reluctance and incompetence of some staff to provide appropriate screening or to engage people with SMI. Liaison is required between those providing potentially diabetogenic or weight promoting antipsychotics, those monitoring mental state, and those providing interventions for CHD risk factors.

The findings of our qualitative study offer a unique insight into the views of service users and NHS staff in relation to the primary prevention of CHD in SMI. To our knowledge, there has been no previous research to address the question of how best to organise clinical services to prevent heart disease in this 'at risk' patient group. Obtaining the views of stakeholder groups is an important first step in developing services that are likely to be acceptable to both service users and providers.

### Study limitations

Our in-depth interviews have explored the views of three important stakeholder groups – i.e. patients with SMI, staff from general practice and staff from CMHTs. However, there are limitations to the conclusions that can be draw from data collected from a relatively small sample recruited from two inner London boroughs. Care therefore should be taken when generalising our findings to other services or settings. The possibility of response bias also exists, in that service users and staff with a particular interest in the topic may have been more likely to agree to participate in an interview. Thus the opinions of those who do not view the prevention of CHD in SMI as a high priority may be under-represented. Nonetheless, the use of qualitative methods has enabled us to obtain a better understanding of the reasons why service users and health professionals hold certain preferences for care delivery.

## Conclusion

Our results suggest that a complex solution is required to this complex problem. Research is required to evaluate the clinical and cost effectiveness of interventions to improve rates of CHD risk factor screening in people with SMI. Such interventions need to straddle primary and secondary care services, be flexible and accessible, and provide effective communication and liaison between relevant health professionals. One or more individuals need to take responsibility for this role and we are currently developing and testing the feasibility and acceptability of a dedicated nurse facilitator who will monitor screening, encourage screening in either primary or secondary care and provide it him/herself if neither option succeeds.

## Competing interests

The author(s) declare that they have no competing interests.

## Authors' contributions

All the authors (CAW, DJPO, IN and MBK) developed the protocol and the interview schedules. CAW conducted and transcribed the qualitative interviews. CAW and DPJO participated in the data analysis and interpretation, and drafted the paper. MBK and IN supervised the research and commented on the drafts of the paper. All authors read and approved the final manuscript.

## Pre-publication history

The pre-publication history for this paper can be accessed here:


